# Characteristics of Refractive Errors in a Population of Adults in the Central Region of Poland

**DOI:** 10.3390/ijerph15010090

**Published:** 2018-01-08

**Authors:** Michal S. Nowak, Piotr Jurowski, Andrzej Grzybowski, Janusz Smigielski

**Affiliations:** 1 Provisus Eye Clinic, 112 Redzinska str., 42-209 Czestochowa, Poland; 2 Department of Ophthalmology and Visual Rehabilitation, Medical University of Lodz, 113 Zeromskiego str., 90-549 Lodz, Poland; p.jurowski@vp.pl; 3 Department of Ophthalmology, University of Warmia and Mazury, 30 Warszawska str., 10-082 Olsztyn, Poland; ae.grzybowski@gmail.com; 4 Department of Statistics, State University of Applied Science in Konin, 1 Przyjazni str., 65-510 Konin, Poland; janusz.smigielski.stat@gmail.com

**Keywords:** myopia, hyperopia, anisometropia

## Abstract

*Background*: To investigate the distribution of refractive errors and their characteristics in older adults from a Polish population. *Methods*: The study design was a cross-sectional study. A total of 1107 men and women were interviewed and underwent detailed ophthalmic examinations, 998 subjects underwent refraction. Myopia was defined as spherical equivalent (SER) refraction ≤−0.5 dioptres (D) and hyperopia was defined as SER ≥+0.5 dioptres (D). *Results*: Among those who were refracted the distribution of myopia and hyperopia was 24.1% (95% CI 21.4–26.7) and 37.5% (95% CI 34.5–40.5), respectively. Myopia decreased from 28.7% in subjects aged 35–59 years to 19.3% in those aged 60 years or older and hyperopia increased from 21.8% at 35–59 years of age to 53.3% in subjects aged ≥60 years. Multiple regression analysis showed decreasing age (OR 0.98, 95% CI 0.96–1.00), female gender (OR 1.87, 95% CI 1.18–2.95) and presence of cataract (OR 2.40, 95% CI 1.24–4.63) were independent risk factors associated with myopia. *Conclusions*: The distribution of refractive errors found in our study is similar to those reported in other Caucasian populations and differs from Asian populations. Myopia was positively associated with younger age, female gender and presence of cataract.

## 1. Introduction

According to the latest reports of World Health Organization (WHO) uncorrected refractive errors are the most common cause of visual impairment worldwide, accounting for 43% of cases and representing an important cause of blindness [1]. Uncorrected refractive errors have also been associated with reduced vision-related quality of life and with loss of independence [2,3]. The estimated global cost of lost productivity due to refractive error vision impairment in 2007 was more than 200 billion United States dollars [4]. Most of this could be eliminated simply with refraction and appropriate vision correction [5,6].

The prevalence of refractive errors has been reported to vary with race, age, gender and geographic regions. Population-based data indicate the prevalence of myopia as being higher in children of Chinese ethnicity; but in Chinese adults the rate of myopia is not much higher than what is found in White adult population [2]. Environmental factors like level of education, occupation, near-work load, time outdoors as a child are also associated with aetiology of refractive errors [2,7,8,9]. The gender differences in the prevalence of refractive errors have been also reported, but many studies have failed to confirm these associations [7,10,11,12,13,14]. 

During the last two decades several studies concerning the prevalence of refractive errors in Asia [12,13,14,15,16,17,18,19], Australia [20,21] and North America [2,9,22,23,24] have been undertaken. However there are very few from Europe and all are from the Western part [7,11,25,26]. Poland is the biggest eastern European country, with a population of 38 million people according to the 2011 national census [27]. Due to a lack of data from Poland and other post-Soviet nations, we conducted an epidemiological survey on a sample population of older adults in the city of Lodz, which results have recently been published [6,28]. The aim of the present study was to investigate the distribution of refractive errors and their characteristics in this population.

## 2. Materials and Methods

### 2.1. Subjects, Eye Examinations and Definitions

The study design was a cross-sectional study. The sampling and recruitment methods for this study have been described in details in our previous papers [6,28]. Sample size for the study was calculated with 99% confidence, within an error bound of 5%. The sample size requirement was 661, as calculated by:

N = Z^2^/4d^2^ (1)

where Z = 2.57 for 99% confidence interval and d = 0.05 for 5% error bound. After allowing for an arbitrary 50% increase in sample size to accommodate possible inefficiencies associated with the sample design, the sample size requirement increased to 991 subjects [6,28]. We decided to define an older adult as person aged ≥35 years because in our previous reports conducted on young males in the military population, we considered young adults as persons aged 18–34 years [29,30]. We used simple systematic sampling to select our study population. In total 14,110 outpatients were examined in the Department of Ophthalmology and Visual Rehabilitation of the Medical University of Lodz in year 2012 and we included into the study every tenth subject aged 35 years and older [28]. Based on age, the study subjects were divided into two groups; group I, aged 35–59 years, and group II, aged 60 years and older. All participants were interviewed and information regarding brief details of the eye conditions, age, sex and socioeconomic status was collected. Comprehensive ophthalmic examination included: distance visual acuity (VA) testing, a cover test, binocular and color vision assessments, intraocular pressure (IOP) measurements as well as slit lamp and indirect ophthalmoscopic evaluation of the anterior and posterior segments and other examinations where needed. Distance visual acuity (VA) was tested monocularly, using a retroilluminated Snellen chart placed at 4 m. Because the present study was a continuation of our previous reports as mentioned earlier, we used the methodology of refraction measurements and definitions of refractive errors from the Polish Army national regulations for ophthalmic examination [29,30]. Autorefraction data were obtained in all study subjects using a Topcon KR 8900 autorefractometer (supplied by Topcon Corporation, Tokyo, Japan). Cycloplegic refraction data were obtained only in eyes presenting with distance visual acuity <20/40 Snellen (0.3 logMAR). Based on this, subjective refraction tests using only spherical or cylindrical glasses were performed to achieve best corrected visual acuity (BCVA). Spherical equivalent (SER) refractive error, defined as sphere plus half cylinder, was applied for myopia and hyperopia calculations. According to the Polish Army regulations myopia was defined as spherical equivalent (SER) refraction ≤−0.5 dioptres (D), hyperopia was defined as SER ≥+0.5 dioptres (D) and emmetropia as SER between −0.5 and +0.5 diopters (D). Astigmatism was considered if the cylinder was ≥0.5 dioptres [29,30]. Anisometropia was defined as difference of SER greater than 1.0 dioptres (D) between the right and the left eyes. The distribution of refractive errors was presented binocularly. If the study subject had one myopic and a fellow hyperopic eye, the refractive error of the eye with larger spherical equivalent was taken into account. Eyes with previous history of cataract surgery, which underwent corneal transplantation and with ocular conditions which precluded autorefraction measurements were excluded from statistical analysis.

For this report the presence of cataract, aphakia or pseudophakia was determined on the slit lamp examination. Glaucoma was diagnosed when characteristic morphological changes of the optic nerve head and retinal nerve fiber layer (RNFL) not related to other ocular disease or congenital anomalies were present, associated with typical glaucomatous visual filed loss. The ocular hypertension (OHT) was diagnosed if the intraocular pressure was elevated with all other ocular findings within normal limits [28,31]. In a few subjects with large media opacities, when results of optic nerve head examinations and visual field were unavailable, glaucoma was diagnosed basing on previous evidence of glaucoma treatment.

Because of the nature of the survey, verbal informed consent was obtained from all study participants. The institutional review board waived the need for written informed consent from the participants, but otherwise the work was conducted in accordance with the provisions of the Declaration of Helsinki for research involving human subjects and was approved by the ethic committee of the Medical University of Lodz (Ethical Approval Code RNN/848/12/KB).

### 2.2. Data Management and Statistical Analysis

A commercially available software STATISTICA v. 10.1 PL (StatSoft Polska, Krakow, Poland) was used to perform all statistical analyses. Age-specific prevalence rates of myopia, hyperopia and astigmatism were calculated in subjects with distance visual acuity <20/40 after cycloplegic refraction. The associations between the distance visual acuity categories as well as refractive errors with the subjects’ age and gender were explored by χ^2^ statistics (*p* < 0.05). Multiple logistic regression statistics were used to investigate the association of myopia and hyperopia with age, gender, socioeconomic status of participants as well as with cataract, glaucoma and ocular hypertension (OHT). All presented confidence intervals (CIs) were 95% CI and odds ratios (ORs) were computed. 

## 3. Results

### 3.1. Subjects

A total of 1107 white subjects aged ≥35 years, most of whom live or have lived in the city of Lodz, in central Poland were enumerated and included into the study. The mean age of the study subjects was 60.4 ± 12.8 years (range, 35–97 years). There were 465 men (42%) and 642 women (58%). According to 2011 national census, our study participants were a fair representation of the population of the city of Lodz in terms of sex distribution (statistical analysis- chi square test: χ^2^ = 3.64, *p* > 0.05) and socioeconomic status [27]. Statistical analysis also revealed that our two age groups did not vary significantly in gender (χ^2^ test *p* = 0.158). Socio-demographic analysis revealed only 31 subjects (2.8%) declared to have no source of income. The number of subjects with no income was significantly higher in age group 35–59 years.

### 3.2. Distribution of Distance Visual Acuity and Refractive Errors

Visual acuity (VA) measurements were obtained in 2214 eyes of 1107 subjects (Table 1). In total 72.5% (95% CI 69.9–75.1) subjects had normal or near normal vision-distance VA of ≥20/40 in both eyes and 27.5% (95% CI 24.8–30.1) had distance VA of <20/40 in worse-seeing eye. There were significant differences in distant visual acuity between the age and gender categories (*p* = 0.01). The number of individuals with better VA was lower, and the number of individuals with worse VA was higher in the age group ≥60 years and in women. After cycloplegic and subjective refractions only 1.8% (95% CI 1.0–2.6) of subjects had best corrected visual acuity (BCVA) of ≤20/200 in both eyes. 

**Figure 1:**
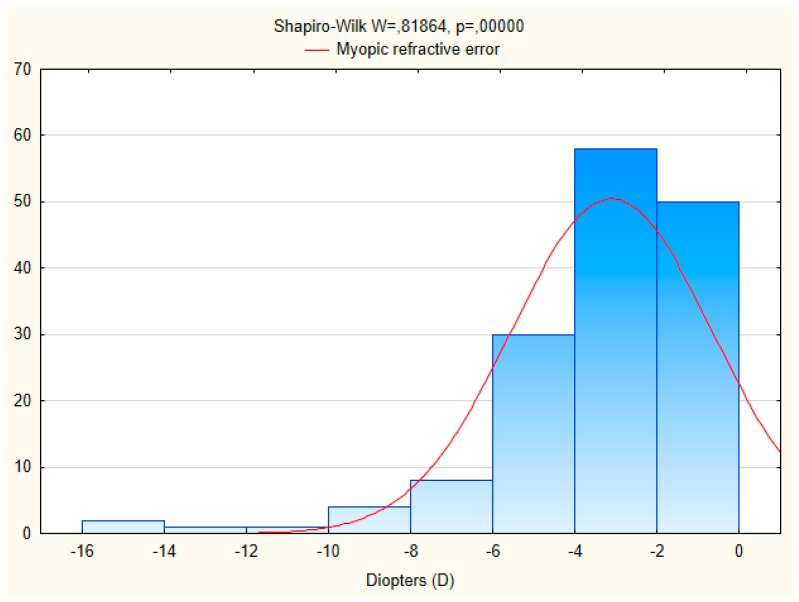
Histogram of myopic refractive error in the researched population.

**Figure 2:**
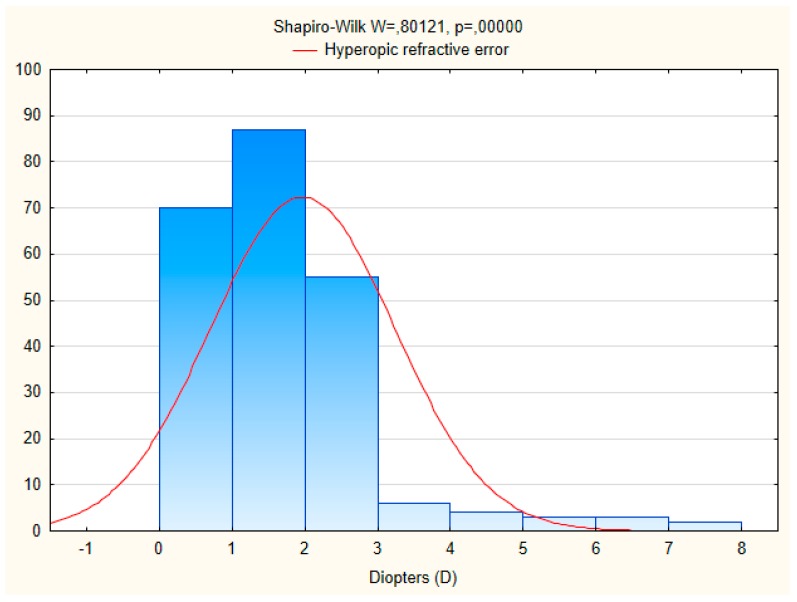
Histogram of hyperopic refractive error in the researched population.

Data on refractive errors were available for 998 individuals (Table 2). Myopia was found in 21.7% of the males and in 25.7% of the women. The distribution of hyperopia and astigmatism was 37.5% and 10.8%, respectively. Gender-specific rates of myopia, hyperopia and astigmatism were statistically significant (χ^2^ test *p* < 0.001). Hyperopia was more common in women (42.0%) and asigmatism in men (13.0%) than in women (9.3%). In age group 60 years and older there was a significant increase in the number of subjects with hyperopia and astigmatism compared to age group 35–59 years; while the number of subjects with myopia decreased with age. The mean spherical equivalent refraction (SER) of myopia and hyperopia was 3.1 ± 2.4 diopters and 2.0 ± 1.3 diopters, respectively. The characteristic of myopic and hyperopic refractive errors obtained with autorefraction is presented on Figure 1 and Figure 2. Anisometropia greater than 1.0 dioptres (D) was found in 9.2% (95% CI 5.5–12.9) of subjects.

### 3.3. Multiple Logistic Regression Modeling

Multivariate logistic regression models were constructed to analyze the risk factors for myopia and hyperopia in this group (Table 3). 

Our analysis showed that hyperopia was significantly associated with age (OR 1.02, 95% CI 1.00–1.04). Myopia was also significantly associated with age (OR 0.98, 95% CI 0.96–1.00) but in opposite direction. After adjusting for all other factors women were more likely to have hyperopia (OR 2.16, 95% CI 1.38–3.38) compared with myopia (OR 1.87, 95% CI 1.18–2.95). The presence of cataract was a significant risk factor for myopia (OR 2.40, 95% CI 1.24–4.63). No association was found between refractive errors and socioeconomic status of our study subjects.

## 4. Discussion

This study describes refractive errors in a group of Polish citizens’ aged 35 years or older, living in the city of Lodz in central Poland. It provides for the first time data concerning the distribution of refractive errors and their characteristics for the region. All of the study participants were white Caucasians and had a demographic composition similar to the 2011 national census population [27], which also supports the findings. Among those who were refracted, the prevalence rate of myopia (SER ≤ 0.5 D) was 24.1% and decreased from 28.7% in subjects aged 35–59 years to 19.3% in those aged 60 years or older. Our results were not far from the results of the epidemiological study on older adults of predominantly European Caucasian origin performed in recent years in Spain—The Segovia Study where myopia prevalence of 25.4% was found [11]. In addition our results were lower than those reported in non-Hispanic whites in the 2005–2008 National Health and Nutrition Examination Survey (NHANES) in the United States and in Japan, South Korea and among Chinese in Singapore [15,19,24,32]. But were higher than those found among older adults in Australia, predominantly of European Caucasian origin, in the Blue Mountains Eye Study and among African-Americans in Barbados, Chinese in Beijing or Taiwan and in studies from Nigeria, Bangladesh and Argentina [9,16,17,20,33,34,35]. All these studies were population based and not hospital based. Comparison of sampling techniques and the prevalence rates of refractive errors in different populations from previously published studies is presented in Table 4. 

Hyperopia (SER ≥ 0.5 D) was the most common refractive error in our study accounting for 37.5% and increased from 21.8% in subjects aged 35–59 years to 53.3% in those aged 60 years and older. High rates of hyperopia prevalence were also found in older British adults in the EPIC-Norfolk Eye Study [25] and in adult Americans in Beaver Dam Eye Study in Wisconsin [22]. The multiple regression analysis showed that increasing age and female gender were significantly associated with hyperopia. Factors associated with myopia were the same but age was associated in opposite direction. Myopia was also positively associated with the presence of any cataract. 

Our findings were in agreement with the results of some previous studies, which demonstrated a decrease of the prevalence of myopia and, simultaneously, an increase in the prevalence of hyperopia with increasing age [3,10,11,23]. The results of other studies also revealed that myopia was associated with higher level of education, professional occupations requiring near-work, less outdoor activities as well as with nuclear lens opacities and ocular dimensions [2,8,10,36]. Some studies showed hyperopia was associated with age, female gender, lower educational level, non-professional occupations and decreased axial length, though their findings were not consistent [9,10,12]. The distribution of astigmatism in our study was higher in men and in age group 60 years and older. Anisometropia greater than 1.0 D was found in 9.2% of subjects, which is comparable with the findings from Singapore, Mongolia and Spain [11,12,37].

Correction of refractive errors across the world is one of the biggest challenges for public health. Although refractive errors cannot be prevented, they can easily be diagnosed and corrected for a relatively small costs. Limitations to the present study included differences in study design and population sampling with possible presence of selection bias. We cannot directly compare our data with other population-based studies. Patients enrolled into the study were solely from our Outpatients Department thus the prevalence of ocular disorders might be higher than in general population. Other limitation was that cycloplegic refraction data were collected only in subjects with VA < 20/40 Snellen (0.3 logMAR).

## 5. Conclusions

In conclusion, this study provides for the first time epidemiologic data on refractive status of individuals aged 35 years and older in Poland. The distribution of refractive errors found in our study is similar to those reported in other Caucasian populations in Western Europe and America, but differs from Asian populations. In our study population myopia was positively associated with younger age, female gender and presence of any cataract. To the best of our knowledge the distribution and characteristic of refractive errors among European Caucasian adults in Eastern Europe have not been previously reported. However, further investigations are needed on a larger, randomly selected populations.

## Figures and Tables

**Table 1:** Distribution of distance visual acuity among our study subjects.

Visual Acuity Category	Right Eyes *n* (%; 95% CI)	Left Eyes *n* (%; 95% CI)		Both Eyes *n* (%; 95% CI)
≥20/40	842 (76.0%; 73.5.5–78.6)	846 (76.4%; 73.9–78.9)		803 (72.5%; 69.9–75.1)
>20/200 <20/40	209 (18.9%; 16.6–21.2)	202 (18.3%; 16.0–20.5)		224 (20.2%; 17.8–22.6) †
≤20/200	56 (5.1%; 3.8–6.3)	59 (5.3%; 4.0–6.6)		80 (7.3%; 5.7–8.7) †
All	1107 (100%)	1107 (100%)		1107 (100%)
	**35–59 Years**		**≥60 Years**	
≥20/40	397 (76.4%; 72.7–80.0)		406 (69.2%; 65.4–72.9)	
>20/200 <20/40	88 (16.9%; 13.7–20.1) †		136 (23.2%; 19.7–26.6) †	
≤20/200	35 (6.7%; 4.6–8.9) †		45 (7.6%; 5.5–9.8) †	
all	520 (100%)		587 (100%)	
χ^2^ test *p* < 0.001				
	**Men**		**Women**	
≥20/40	358 (77.0%; 73.2–80.8)		445 (69.3%; 65.7–72.9)	
>20/200 <20/40	76 (16.3%; 13.0–19.7) †		148 (23.1%; 19.8–26.3) †	
≤20/200	31 (6.7%; 4.4–8.9) †		49 (7.6%; 5.6–9.7) †	
all	465 (100%)		642 (100%)	
χ^2^ test, *p* = 0.01				

† in worst eye.

**Table 2:** Distribution of refractive errors in a researched group.

Refractive Error	35–59 Years (*n*; %; 95% CI)	≥60 Years (*n*; %; 95% CI)	Men (*n*; %; 95% CI)	Women (*n*; %: 95% CI)	Totally (*n*; %; 95% CI)
Emmetropia (>−0.5 D, <+0.5 D, SE)	214 (42.7%; 38.4–47.0)	62 (12.5%; 9.6–15.4)	142 (34.2%; 29.6–38.8)	134 (23.0%; 19.6–26.4)	276 (27.6%; 24.9–30.4)
Myopia (≤−0.5 D, SE)	144 (28.7%; 24.8–32.7)	96 (19.3%; 15.8–22.8)	90 (21.7%; 17.7–25.6)	150 (25.7%; 22.2–29.3)	240 (24.1%; 21.4–26.7) †
Hyperopia (≥+0.5 D, SE)	109 (21.8%; 18.1–25.4)	265 (53.3%; 48.9–57.7)	129 (31.1%; 26.6–35.5)	245 (42.0%; 38.0–46.0)	374 (37.5%; 34.5–40.5) †
Astigmatism (≥0.5 D, Cyl)	34 (6.8%; 4.6–9.0)	74 (14.9%; 11.8–18.0)	54 (13.0%; 9.8–16.2)	54 (9.3%; 6.9–11.6)	108 (10.8%; 8.9–12.7) †
All	501 (100%)	497 (100%)	415 (100%)	583 (100%)	998 (100%)

χ^2^ test *p* < 0.001; † at least in one eye.

**Table 3:** Multiple logistic regression models of the risk factors for myopia and hyperopia.

Variables	Myopia ≤ 0.5 D	Hyperopia ≥ 0.5 D
	OR, 95% CI, *p* Value	OR, 95% CI, *p* Value
Age, per year increase	0.98 (0.96–1.00); *p* = 0.023	1.02 (1.00–1.04); *p* = 0.046
Women vs. men	1.87 (1.18–2.95); *p* = 0.007	2.16 (1.38–3.38); *p* = 0.001
Any cataract	2.40 (1.24–4.63); *p* = 0.009	1.68 (0.96–2.96); *p* = 0.070
Glaucoma and ocular hypertension (OHT)	0.36 (0.11–1.19); *p* = 0.094	0.52 (0.22–1.23); *p* = 0.136
Socioeconomic status	1.21 (0.35–4.14); *p* = 0.766	1.87 (0.62–5.63); *p* = 0.264

**Table 4:** Comparison of sampling techniques and the prevalence rates of refractive errors in different populations from previously published studies.

Epidemiological Study	Sampling Technique	Age Group (Years)	Myopia (%)	Hyperopia (%)	Astigmatism (%)	Anisometropia (%)
The Beaver Dam Eye Study (USA) [22] †	a door to door census	≥43	26.2	49.0	NA	NA
The Blue Mountains Eye Study (Australia) [20] †	a door to door census	≥49	15.5	56.6	NA	NA
The Tajimi Study (Japan) [15] †	random sampling	≥40	41.8	27.9	54.0	15.1
The Gutenberg Health Study (Germany) [7] †	random sampling	≥35	35.1	32.8	32.3	13.5
The Barbados Eye Study (Barbados) [9] †	random sampling	≥40	21.9	46.9	NA	NA
The Singapore Indian Eye Study (Singapore) [12] †	age-stratified random sampling	≥40	28.0	35.9	54.9	9.8
The Segovia Study (Spain) [11] ‡	age-stratified random sampling	≥40	25.4	43.6	53.5	12.3
The Yazd Eye Study (Iran) [18] ‡	multistage random cluster sampling	≥40	36.5	20.6	53.8	11.9
Korean National Health and Nutrition Examination Survey (South Korea) [19] §	multistage stratified cluster random sampling	≥20	48.1	24.2	34.0	NA
The Nigerian National Blindness and Visual Impairment Study (Nigeria) [33] †	multistage stratified cluster random sampling	≥40	16.2	50.7	63.5	NA
The National Blindness and Low Vision Survey of Bangladesh (Bangladesh) [34] †	cluster sampling and a door to door enumeration	≥30	22.1	20.6	32.4	7.5
The Shihpai Eye Study (Taiwan) [17] †	random sampling and a door to door enumeration	≥65	19.4	59.0	74.0	21.8

† Myopia (<−0.5 D), Hyperopia (>+0.5 D), Astigmatism (>0.5 cyl D), Anisometropia (>1.0 D). ‡ Myopia (<−0.5 D), Hyperopia (>+0.5 D), Astigmatism (>0.5 cyl D), Anisometropia (≥1.0 D). § Myopia (<−0.5 D), Hyperopia (>+0.5 D), Astigmatism (>1.0 cyl D).
